# Maternal and neonatal outcomes in women undergoing trial of labor after cesarean (TOLAC) compared with elective repeat cesarean section (ERCS): a cohort study

**DOI:** 10.1186/s12884-025-08447-6

**Published:** 2025-12-01

**Authors:** Konstantinos Chatzistergiou, Jean-Baptiste Chanier, Richard Paul-Dehlinger, Gregory Bierry, Bruno Renevier, Simon Crequit

**Affiliations:** Department of Gynecology and Obstetrics, Intercommunal Hospital Center André Grégoire, 56 Boulevard de la Boissière, Montreuil, 93100 France

**Keywords:** Trial of labor after cesarean, Programmed cesarean section, Uterine rupture, Postpartum hemorrhage, Neonatal outcomes

## Abstract

**Background:**

Trial of labor after cesarean (TOLAC) is often recommended as an alternative to elective repeat cesarean section (ERCS) in women with a single prior cesarean section. This retrospective cohort study aimed to compare obstetrical outcomes between TOLAC and ERCS in a French hospital setting.

**Methods:**

Women with a single prior cesarean delivery, cephalic presentation, and term pregnancies (≥ 37 weeks) between 2019 and 2024 were included. Women were analyzed according to their initially intended mode of delivery (TOLAC or ERCS), irrespective of the final delivery route, to reflect a real-world, decision-based clinical approach. Primary outcomes were uterine rupture, severe postpartum hemorrhage (PPH), and neonatal intensive care unit (NICU) admission. Statistical methods included chi-square tests, Student’s t-tests, and Firth’s penalized logistic regression for rare events.

**Results:**

In total, 2,424 women were included, with 2,146 undergoing TOLAC and 278 undergoing ERCS. Severe PPH occurred less frequently in the TOLAC group (5.6%) than in the ERCS group (10.4%, *p* = 0.002), with adjusted analysis confirming a significant reduction in risk (adjusted OR: 0.45, 95% CI: 0.31–0.69, *p* < 0.001). NICU admissions were also lower in the TOLAC group (6.5% vs. 13.3%, *p* < 0.001; adjusted OR: 0.45, 95% CI: 0.31–0.69, *p* < 0.001). Uterine rupture rates were low and showed no significant difference in unadjusted analysis (1.7% TOLAC vs. 1.1% ERCS, *p* = 0.622), though adjusted models indicated an increased risk with TOLAC (adjusted OR: 2.08, 95% CI: 1.24–3.58, *p* = 0.005).

**Conclusion:**

The findings suggest that obstetrical practice concerning TOLAC, in a French tertiary care maternity unit, is associated with lower rates of severe PPH and NICU admission compared to ERCS, with a slightly increased risk of uterine rupture. These results support offering TOLAC as a safe and beneficial option for eligible women.

## Background

Cesarean delivery rates have risen substantially worldwide, with global estimates showing a marked increase from 7% in the 1990s to over 21% in 2021 [1]. This trend has provoked significant interest in trial of labor after cesarean (TOLAC) as a strategy to reduce repeat cesareans, which are associated with increased maternal and neonatal morbidity and complications in subsequent pregnancies [2, 3]. While cesarean delivery can be lifesaving when medically indicated, it can also contribute to potential risks.

TOLAC offers several potential benefits, including reduced surgical morbidity, shorter recovery time, and lower risks of complications in future pregnancies [4]. However, concerns persist regarding rare but severe complications, such as uterine rupture and emergency cesarean Sect [5]. Elective repeat cesarean section (ERCS), though often perceived as safer in certain contexts, carries its own risks, including surgical morbidity and respiratory complications in neonates [6].

Guidelines from the American College of Obstetricians and Gynecologists (ACOG) [7], the National Institute for Health and Care Excellence (NICE) [8], and the French College of Gynecologists and Obstetricians (CNGOF) [9] advocate TOLAC as the preferred approach in carefully selected patients. However, data comparing maternal and neonatal outcomes between TOLAC and ERCS remain essential to inform clinical decision-making. This study aims to communicate the obstetrical outcomes of TOLAC compared to ERCS, focusing on severe postpartum hemorrhage, uterine rupture, and neonatal NICU admission.

## Methods

This retrospective cohort study was conducted at a single tertiary maternity unit (CHI Montreuil) and included all women with a single previous cesarean section, a singleton pregnancy in cephalic presentation, who delivered after 37 weeks of gestation between January 2019 and December 2024. Exclusion criteria were women with more than one previous cesarean sections, multiple gestations, non-cephalic presentations, fetal demise, major fetal anomalies, or unknown mode or timing of delivery. Participants were categorized into two groups according to their initially intended mode of delivery: trial of labor after cesarean (TOLAC) or elective repeat cesarean section (ERCS). Women who had planned an ERCS but went into spontaneous labor before the scheduled date and ultimately delivered vaginally remained classified in the ERCS group. Likewise, women who initially intended TOLAC but underwent a cesarean delivery before labor for maternal or fetal indications were retained in the TOLAC group. This approach was chosen to reflect real-life clinical decision-making and management.

High medical risk before pregnancy was defined by a history of one or more of the following conditions: history of cardiac disease, hypertension, diabetes, venous thrombosis, pulmonary embolism, Graves’ disease, asthma, homozygous sickle cell anemia, thrombocytopenia, coagulation disorder, a rare or systemic disease, nephropathy, HIV infection, psychiatric disease. High obstetrical risk before pregnancy was defined by a history of one or more of the following conditions: pre-eclampsia, fetal growth restriction, preterm delivery, fetal death, or neonatal death.

Current pregnancy was considered as high-risk pregnancy at the presence of one of the following conditions: suspected small for gestational age (SGA), fetal growth restriction (FGR), preeclampsia, gestational diabetes, or suspected macrosomia. The primary outcomes were uterine rupture (complete division of all three layers), severe postpartum hemorrhage (defined as blood loss > 1500 mL and maternal ICU admission), and NICU admission. Indications for cesarean section were categorized into five main groups. Fetal indication referred to cesarean sections performed before the onset of labor due to evidence of antenatal fetal compromise. Labor obstruction referred to labor prolongation or arrest. Non-reassuring fetal heart rate (FHR) referred to abnormal intrapartum cardiotocography patterns during labor. Maternal indication referred to cesarean deliveries performed before or during the onset of labor due to evidence of maternal health compromise. Finally, maternal desire included cesarean sections performed upon maternal request.

Due to the rare occurrence of the studied outcomes, Firth’s penalized-likelihood logistic regression was employed to address potential complete or quasi-complete separation issues that can arise in standard logistic regression with rare events [10, 11]. This method reduces small-sample bias in maximum likelihood estimation and provides more reliable estimates when analyzing rare events or when the number of events per predictor variable is low [12].

For uterine rupture and postpartum hemorrhage analyses, the models were adjusted for established risk factors identified in previous studies: maternal age, BMI, inter-pregnancy interval ≤ 12 months, birthweight > 4000 g, prior vaginal delivery, and high-risk pregnancy status. The NICU admission model included a reduced set of covariates (maternal age, BMI and high-risk pregnancy status) based on previous literature on neonatal outcomes.

Statistical power for these analyses was assessed using a bootstrap-based simulation approach with 1,000 resamples of the original dataset. For each bootstrap iteration, the full Firth’s logistic regression model was refitted, and power was calculated as the proportion of replicates where the p-value for the primary exposure (trial of labor after cesarean) was below the predetermined alpha level of 0.05. This approach accounts for the actual data structure, sample size, and effect sizes present in the study population while maintaining the complex covariate relationships.

The analyses revealed powers of 70.4%, 70.7%, and 71.2% to detect the observed associations between trial of labor after cesarean and uterine rupture, severe postpartum hemorrhage, and NICU admission, respectively, at the 0.05 significance level. All simulations achieved complete convergence across the 1,000 iterations. R software (R Development Core Team (2008), version 4.2.0) was used for all statistical analysis.

## Results

From a total of 19,451 deliveries occurring between 2019 and 2024, a subset of 2,424 deliveries met the inclusion criteria among which the majority; 2,146 (88.5%) underwent TOLAC, while 278 (11.5%) underwent ERCS. Concerning demographic characteristics, women in the ERCS group were older (median age: 34.25 vs. 33.13 years, *p* = 0.001) and more likely to have high-risk pregnancies (57.6% vs. 49.2%, *p* = 0.010). Maternal BMI was comparable between groups. Maternal origin distribution was diverse with relatively even representation across ethnic groups: Caucasian, North African, Sub-Saharan African, and other categories. A significantly higher proportion of women in the TOLAC group had a history of vaginal delivery (47.9% vs. 27.7%; *p* < 0.001) but also lower rates of high-risk pregnancy (57.6% vs. 49.2%; *p* < 0.01). There was no difference between groups concerning inter-pregnancy interval shorter than 6 months or 12 months (Table 1).

**Table 1:** Demographics

	ERCS	TOLAC	*p*
n	278	2146	
maternal age (median [IQR])	34.25 [30.80, 37.56]	33.13 [29.48, 36.62]	0.001
maternal BMI (median [IQR])	26.69 [23.44, 31.25]	27.12 [23.68, 30.48]	0.648
maternal origin (%)			0.504
caucasian	86 (30.9)	605 (28.2)	
north africa	75 (27.0)	571 (26.6)	
others	38 (13.7)	268 (12.5)	
Sub-Saharan Africa	79 (28.4)	702 (32.7)	
prior vaginal delivery (%)	77 (27.7)	1029 (47.9)	< 0.001
high obstetrical risk level = low risk (%)	226 (81.3)	1709 (79.6)	0.569
high medical risk level = low risk (%)	226 (81.3)	1847 (86.1)	0.042
high risk pregnancy (%)	160 (57.6)	1055 (49.2)	0.010
gestational diabetes (%)	114 (41.0)	815 (38.0)	0.362
pprom (%)	12 (4.3)	115 (5.4)	0.555
inter-pregnancy interval < 6 months (%)	13 (4.7)	56 (2.6)	0.079
inter-pregnancy interval < 12 months (%)	33 (11.9)	197 (9.2)	0.183

*IQR* interquartile range; pprom: preterm premature rupture of membranes

In the TOLAC group, half of the patients had spontaneous labor, one fourth had cervix preparation by Folley catheter and the rest labor induction by oxytocin.

Concerning mode of delivery, in the ERCS group 84% of patients were finally operated as planned and 16% of patients required an emergency cesarean section for fetal or maternal indication. In the TOLAC group, 71% of patients delivered vaginally and 24% by cesarean section during labor (Table 2).

Concerning the primary outcomes of the study, severe postpartum hemorrhage occurred in 10.4% of the ERCS group compared to 5.6% in the TOLAC group (*p* = 0.002). Adjusted analysis confirmed a significant reduction in severe PPH risk associated with TOLAC (adjusted OR: 0.45, 95% CI: 0.31–0.69, *p* < 0.001). Postpartum hemorrhage (> 500 ml) rates were also statistically more frequent in the ERCS group (18.7% versus 12.4%; p 0.005) (Table 2).

Uterine rupture rates were low, with no significant difference observed in unadjusted analysis (1.7% in the TOLAC group vs. 1.1% in the ERCS group, *p* = 0.622). However, adjusted models indicated increased odds of uterine rupture in the TOLAC group (adjusted OR: 2.08; 95% CI: 1.24–3.58; *p* = 0.005) (Table 2). The absolute risk reduction (ARR) associated with ERCS was 0.6% (95% CI − 0.7% to 1.9%), corresponding to a number needed to treat (NNT) of 167 (95% CI 52 to ∞) to prevent one case of uterine rupture.

**Table 2:** Maternal outcomes

	ERCS	TOLAC	*p*
n	278	2146	
study groups (%)			< 0.001
foley cathether	0 (0.0)	534 (24.9)	
labor induction	0 (0.0)	482 (22.5)	
ERCS	278 (100.0)	0 (0.0)	
spontaneous labor	0 (0.0)	1130 (52.7)	
maternal delivery mode (%)			< 0.001
cesarean section during labor	30 (10.8)	520 (24.2)	
emergency cesarean section before labor	16 (5.8)	94 (4.4)	
ERCS	232 (83.5)	0 (0.0)	
vaginal delivery	0 (0.0)	1532 (71.4)	
maternal cesarean section indication (%)			< 0.001
fetal indication	1 (0.4)	38 (6.2)	
labor obstruction	0 (0.0)	315 (51.3)	
maternal desire	261 (93.9)	0 (0.0)	
maternal indication	6 (2.2)	124 (20.2)	
non reassuring intrapartum FHR	10 (3.6)	137 (22.3)	
maternal blood loss (median [IQR])	300.00 [200.00, 371.22]	200.00 [100.00, 300.00]	< 0.001
post partum hemmorhage (%)	52 (18.7)	266 (12.4)	0.005
severe post partum hemmorhage (%)	29 (10.4)	120 (5.6)	0.002
uterine rupture (%)	3 (1.1)	36 (1.7)	0.622

*ERCS* Elective repeat cesarean section

Neonatal NICU admission rates were higher in the ERCS group (13.3%) compared to the TOLAC group (6.5%, p<0.001), with adjusted analysis confirming the benefit (adjusted OR: 0.45, 95% CI: 0.31–0.69, p<0.001). Rates of neonatal weight higher than 4000mg, cord blood pH 7 and 5 minute Apgar scores <7, showed no difference between groups. No neonatal death was documented (Table 3).

**Table 3:** Neonatal outcomes

	ERCS	TOLAC	p
n	278	2146	
neonate birthweight (median [IQR])	3225.00 [2820.00, 3578.75]	3300.00 [3000.00, 3640.00]	0.005
neonate term birth (median [IQR])	39.00 [38.00, 39.00]	39.00 [39.00, 40.00]	< 0.001
birthweight > 4000 g (%)	31 (11.2)	176 (8.2)	0.123
neonate cord blood pH < 7 (%)	2 (0.7)	26 (1.2)	0.671
5 min Apgar score < 7 (%)	4 (1.4)	38 (1.8)	0.877
NICU admission (%)	37 (13.3)	140 (6.5)	< 0.001

*IQR* Interquartile range; NICU: neonatal intensive care unit

## Discussion

This single-center study evaluates maternal and neonatal outcomes of TOLAC compared to ERCS, in a French tertiary care maternity unit. Lower rates of postpartum hemorrhage and NICU admissions were observed in the TOLAC group. Uterine rupture rates were low, with a significant difference observed in adjusted models. These results provide useful insights of obstetrical practice in a French healthcare context.

### Integration with existing knowledge

####  Uterine rupture

The findings of the present study are consistent with existing literature, which consistently reports a higher risk of uterine rupture with TOLAC compared to ERCS, although absolute rates remain low. The wide confidence interval around the absolute risk reduction (ARR) in our analysis reflects both the rarity of uterine rupture and the limited number of events, suggesting that while ERCS may offer a protective effect, the true magnitude of benefit remains uncertain. Recent studies estimate the risk of uterine rupture during TOLAC to range from 0.24% [13] to 1.2% [14], while the American College of Obstetricians and Gynecologists (ACOG) cites an estimated risk of 0.7% [7]. A large U.S. population-based cross-sectional study analyzing over 270,000 singleton pregnancies with one prior cesarean reported a uterine rupture rate of 0.35% in women undergoing TOLAC. Similarly, Guise et al. [3], in a meta-analysis of 47,202 patients, found a uterine rupture risk of 0.47% (95% CI: 0.28–0.77%) for TOLAC, with a relative risk of 20.74 (95% CI: 9.77–44.02, *p* < 0.001) compared to elective cesarean. Landon et al. [5], in a large prospective cohort across 19 U.S. academic medical centers, reported a uterine rupture rate of 0.7% for TOLAC in a population of 17,898 TOLAC and 15,801 ERCS deliveries. The relatively higher rupture rate observed in our study may be explained by the inclusion of older patients, a lower prevalence of prior vaginal deliveries, and a higher incidence of macrosomic neonates compared with previously published articles - all recognized risk factors for uterine rupture.

#### Post partum hemorrhage

Our study found a significantly lower risk of severe postpartum hemorrhage (PPH) in the TOLAC group (5.6%) compared to ERCS (10.4%, *p* = 0.002), with an adjusted OR of 0.45 (95% CI: 0.31–0.69, *p* < 0.001). This trend is supported by Crowther et al. [15], which reported PPH rates of 2.4% in TOLAC vs. 0.8% in ERCS (*p* = 0.011), and Rossi & D’Addario [16], which found PPH in 3.1% for successful VBAC, 4.0% for ERCS, and 17.0% for failed TOLAC (*p* < 0.001). On the contrary, Guise et al. [3] did not show statistically significant difference while Studsgaard et al. [17] (prospective cohort, *n* ≈ 1,800) reported a fivefold increase in hemorrhage risk in TOLAC (*p* < 0.05).

In the current study, the higher incidence of PPH in the ERCS group may be explained by surgical factors, including longer operative times, dissection through scarred tissues, and an increased risk of vascular injury. Moreover, the high rate of successful vaginal births (73%) in the TOLAC group likely contributed to a lower overall risk of hemorrhage. Differences in PPH definitions across studies may also account for some variability in reported rates, as our analysis defined severe hemorrhage as bleeding estimated as more than 1500 mL, whereas other authors use different thresholds. Institutional protocols regarding labor management, uterotonic administration, and transfusion practices may further influence these outcomes.

#### NICU admission

In our study, NICU admissions were significantly lower in the TOLAC group (6.5%) compared to ERCS (13.3%, *p* < 0.001; aOR 0.45, 95% CI: 0.31–0.69). This finding aligns with Kamath et al. [18], a retrospective cohort study (*n* ≈ 2,400) reporting fewer NICU admissions in TOLAC (4.9%) vs. ERCS (9.3%, *p* = 0.025), and with Shi et al. [19], a nationwide cohort of nearly 5 million births, which found a slightly lower NICU admission rate in TOLAC (9.1%) vs. ERCS (9.5%; aOR 0.97, 95% CI: 0.95–0.98). In contrast, Crowther et al. [18] (RCT, *n* = 2,345) and Studsgaard et al. [20] reported significantly higher NICU admissions in TOLAC (5.2% vs. 3.0% and 10.5% vs. 5.7%, respectively). Litwin et al. [20], a large U.S. population-based study (*n* ≈ 1 million), also found increased NICU admissions after TOLAC (aOR 1.12, 95% CI: 1.09–1.16), particularly in cases of uterine rupture (OR 5.95). These discrepancies may reflect variation in TOLAC success rates, induction practices, NICU admission thresholds, and underlying population risks.

#### Strengths and limitations

This current study is based on a well-defined cohort with clear inclusion criteria and robust clinical data. The diversity of origin of included population enhances the study’s generalizability and reduces potential ethnic bias in clinical outcomes while the grouping approach mirrors the intent-to-treat principle by evaluating outcomes based on the initial delivery plan. The high vaginal delivery rate suggests that effective candidate selection and labor management can result in optimal obstetric outcomes without increasing maternal or neonatal risk. However, several limitations should be acknowledged. The retrospective design limits causal inference and may introduce selection bias, particularly in decisions regarding mode of delivery. Although multivariable adjustments were applied, residual confounding cannot be excluded. Additionally, the single-center nature of the study may limit generalizability to other settings with different clinical practices or patient profiles.

## Conclusion

In this retrospective cohort study, trial of labor after cesarean (TOLAC) was associated with lower risks of severe postpartum hemorrhage and NICU admission compared to elective repeat cesarean section (ERCS), despite a slightly higher incidence of uterine rupture. These findings reinforce the safety and maternal–neonatal benefits of TOLAC when applied to well-selected patients within a structured obstetric environment.

## Data Availability

The datasets used and/or analyzed during the current study are available from the corresponding author on reasonable request.

## Abbreviations

ACOG: American College of Obstetricians and Gynecologists
ARR: Absolute Risk Reduction
aOR: Adjusted Odds Ratio
BMI: Body Mass Index
CI: Confidence Interval
CNGOF: Collège National des Gynécologues et Obstétriciens Français
ERCS: Elective Repeat Cesarean Section
FHR: Fetal Heart Rate
FGR: Fetal Growth Restriction
IQR: Interquartile Range
ICU: Intensive Care Unit
NICU: Neonatal Intensive Care Unit
NNT: Number Needed to Treat
OR: Odds Ratio
PPH: Postpartum Hemorrhage
RCT: Randomized Controlled Trial
SGA: Small for Gestational Age
TOLAC: Trial of Labor After Cesarean
VBAC: Vaginal Birth After Cesarean
